# Cognitive effects of transcranial direct current stimulation combined with working memory training in fibromyalgia: a randomized clinical trial

**DOI:** 10.1038/s41598-018-30127-z

**Published:** 2018-08-20

**Authors:** Vinicius Souza dos Souza dos Santos, Maxciel Zortea, Rael Lopes Alves, Cátia Cilene dos Santos Naziazeno, Júlia Schirmer Saldanha, Sandra da Conceição Ribeiro de Carvalho, António Jorge da Costa Leite, Iraci Lucena da Silva Torres, Andressa de Souza, Prisla Ücker Calvetti, Felipe Fregni, Wolnei Caumo

**Affiliations:** 1 Post-graduation Program in Medicine: Medical Sciences, Federal University of Rio Grande do Sul (UFRGS), Porto Alegre, Brazil; 2 Laboratory of Pain & Neuromodulation, Clinical Hospital of Porto Alegre (HCPA), Porto Alegre, Brazil; 3 Graduation Program in Psychology, UFRGS, Porto Alegre, Brazil; 4 Laboratory of Neurophysiology, University of Minho, Braga, Portugal; 5 Neuromodulation Center, Spaulding Rehabilitation Hospital, Harvard Medical School, Boston, United States; 6 Post-Graduation Program in Biological Sciences, Physiology, UFRGS, Porto Alegre, Brazil; 7 Pharmacology of Pain and Neuromodulation: Pre-clinical Investigations, UFRGS, Porto Alegre, Brazil; 8 Post-Graduation Program in Health and Human Development, La Salle University, Philadelphia, United States; 9 Pain and Palliative Care Service at Hospital de Clínicas de Porto Alegre (HCPA), Porto Alegre, Brazil

## Abstract

Cognitive dysfunction in fibromyalgia has been reported, especially memory. Anodal transcranial direct current stimulation (tDCS) over the dorsolateral prefrontal cortex (DLPFC) has been effective in enhancing this function. We tested the effects of eight sessions of tDCS and cognitive training on immediate and delayed memory, verbal fluency and working memory and its association with brain-derived neurotrophic factor (BDNF) levels. Forty females with fibromyalgia were randomized to receive eight sessions of active or sham tDCS. Anodal stimulation (2 mA) was applied over the DLPFC and online combined with a working memory training (WMT) for 20 minutes. Pre and post-treatment neurocognitive tests were administered. Data analysis on deltas considering years of education and BDNF as covariates, indicated active-tDCS + WMT significantly increased immediate memory indexed by Rey Auditory Verbal Learning Test score when compared to sham. This effect was dependent on basal BDNF levels. In addition, the model showed active stimulation increased orthographic and semantic verbal fluency scores (Controlled Oral Word Association Test) and short-term memory (Forward Digit Span). The combination of both techniques seemed to produce effects on specific cognitive functions related to short-term and long-term episodic memory and executive functions, which has clinical relevance for top-down treatment approaches in FM.

## Introduction

Fibromyalgia (FM) is a chronic pain condition with 2 to 5% prevalence in general population, being more frequent in women^1,2^. It comprises widespread chronic pain, fatigue, depression, anxiety, disrupted sleep and other somatic complaints, as well as impaired cognition^3^. In general, the most frequent complains related to cognitive aspects include a poorer recall, difficulty with concentration and attention. Despite the pathophysiology of FM is not completely understood, an imbalance in the excitatory/inhibitory central nervous system (CNS) has being considered^4^. This imbalance comprises a phenomenon of central sensitization syndrome (CCS). The CSS is an amplification of neural signaling within the central nervous system associated with hypersensitivity to pain^5^. In fact, the CCS includes psychological distress, sleep disturbance, allodynia and hyperalgesia^6^. The impaired sustained attention was associated positively with deep-tissue hyperalgesia and deficient conditioned pain modulation^7^. Furthermore, a higher score in the Central Sensitization Inventory (CSI) in chronic pain was positively correlated with level of dysfunction in the descending pain modulatory system, as well as with higher levels of serum brain-derived neurotrophic factor (BDNF)^8^.

Although multiple mechanisms of synaptic plasticity are involved in the CCS, the BDNF has a central role in strengthening glutamatergic synapses, while it weakens GABAergic synapses. The increase of this neurotrophic factor inverts the polarity of GABA currents in dorsal horn neurons^9^. Also, convergent pieces of evidence suggest that BDNF is essential for maintaining the network activity in the prefrontal cortex (PFC)^10^. This region has neurons with intrinsic properties that allow them to initiate, maintain and terminate sustained non-adapting firing. The PFC is provided of extensive dopaminergic projections and other inputs for tuning the state of a network sensible to detect triggers and to initiate activity. Due to the fact that this region is responsible for many functions, it has been extensively used as a target for cognitive enhancement approaches via transcranial direct current stimulation (tDCS)^11–13^. Specifically, the dorsolateral prefrontal cortex (DLPFC) has been targeted for anodal stimulation due to its functional role in updating and maintaining goal-directed representations for context information (which is necessary for episodic memory) and task-related demands^14^.

Accordingly, the anodal tDCS applied to the DLPFC improved working memory (WM) learning curves in healthy adults, who trained on a visual/spatial and verbal adaptive n-Back Task^15^. Similarly, two studies with healthy subjects showed that the anodal stimulation on the left DLPFC improved the WM performance^16^. More robust effects have been reported with the combination of stimulation and a cognitive task^11^. One of the most used tests for working memory training is with the n-Back Task. Andrews *et al*.^11^ found that the tDCS associated with a WM task produced a better performance in another equivalent task applied at a later time. Additionally, recently our research group showed an additive effect of tDCS on DLPFC combined with a task that induces the activation of inhibitory control pathways in FM^13^. The study also shows that the combination of interventions improved performance of attention networks associated with an increase in pain threshold. We hypothesize tDCS may modulate prefrontal circuits, enhancing tolerance and minimizing the emotional component of pain experience. However, there is a gap in terms of exploring baseline neuroplasticity characteristics that could be related to tDCS’s effect on the DLPFC combined with a WM training. Moreover, multiples sessions of this combined treatment may have advantages over a singular session.

In this explanatory trial, we aimed to test if a treatment with active-tDCS combined with a working memory training (WMT) would increase immediate and delayed memory scores, as well as working memory, verbal fluency and divided attention capacity, when compared to sham-tDCS + WMT. We also aimed to test if the treatment effect is dependent on the serum BDNF levels. We hypothesize that neuroplasticity state measured by BDNF has a modulatory role for the effect of tDCS in cognitive performance. In other words, the higher the BDNF serum levels, the larger the anodic tDCS effects on memory and the other cognitive functions.

## Results

### Demographic and clinical characteristics

Thirty-nine patients completed the study (one patient had dropout from the a-tDCS group due to a leg injury). Clinical and demographic characteristics of the sample according to the intervention group are shown in Table 1.

**Table 1:** Demographic and Clinical characteristics (n = 40).

	Active-tDCS (n = 20)	Sham-tDCS (n = 20)	*p*
***Demographic***			
Age (years)	49.15 (8.43)	50.05 (11.19)	0.77
Body weight (Kg)	28.47 (4.18)	27.73(5.20)	0.62
Years of education	10.60(4.36)	10.75(2.86)	0.89
***Clinical***			
Clinical comorbidity (yes /%)	11(55)	15 (75)	0.18
Hypertension (n/%)	6 (30)	7(35)	
Hypothyroidism (n/%)	1 (5)	3(15)	
Asthma (n/%)	1 (5)	2(10)	
Gastritis (n/%)	2 (10)	0(0)	
Diabetes(n/%)	1 (5)	0(0)	
Other (n/%)	0(0)	3(15)	
Beck Depression Inventory – BDI – II	24(9.84)	28(12.96)	0.22
Brazilian Portuguese version of the Pain Catastrophizing Scale (BP-PCS)	30.65(11.89)	31.30(14.91)	0.88
Fibromyalgia Impact questionnaire(FIQ)	63.43(18.29)	66.16(15.31)	0.61
**State-Trait Anxiety Inventory – STAI**			
STAI – State	33.45(6.31)	34.30(7.61)	0.70
STAI – Trait	25.25(4.64)	26.85(6.17)	0.36
Brazilian Profile of Chronic Pain: Screening (B-PCP:S)	69.01(14.15)	70.57(16.10)	0.76
Pittsburgh Sleep Quality Index – PSQI	12.05(4.34)	11.60(4.07)	0.73
Alcohol Consumption (yes/%)	8(40)	6(30)	0.50
Smoking (yes/%)	6(30)	6(30)	1
***Biochemical***			
Serum BDNF	28.70(12.43)	30.41(12.48)	0.67
*Measures of pain*			
Pain score on VAS (0 to100 cm)	7.30(1.66)	7.21(1.66)	0.86
QST: Heat Pain threshold	33.13(1.13)	33.19(1.05)	0.85
QST: Pain tolerance	44.29(3.19)	44(3.0)	0.76
Psychiatric disorder according to the MINI *			
Major depressive episode	11(55%)	16(80%)	0.91
Major depressive episode with dysthymia	7(35%)	6(30%)	0.73
Maniac-depressive disorder	3(15%)	3(15%)	1
Post-traumatic stress disorder	3(15%)	3(15%)	1
Generalized anxiety disorder	6(30%)	9(45%)	0.32
***Medication***			
Analgesic use (yes/%)	12(60)	10(50)	0.52
>4 times a week in the last 3 months t (n/%)	5(25)	7(35)	0.24
<4 times a week (n/%)	7(35)	3(15)	0.36
Aminophen/Dipirone(n/%)	3 (15)	5 (25)	
Non-steroidal anti-inflammatory drugs(n/%)	9 (45)	5 (25)	
Central nervous system active medication(yes/%)	16(80)	16(80)	
Antidepressant (n/%)	9(45)	10(50)	
Anticonvulsant (n/%)	5 (25)	4(20)	
Benzodiazepine (n/%)	2 (10)	2(10)	

Notes. QST = Quantitative Sensory Testing; VAS: visual analog scale; BDNF = Brain-derived neurotrophic factors. Values are given as mean (standard deviation) or frequency (%). Independent samples t-Tests for mean values and Chi-Squared or Fisher’s tests for frequency values. *Most frequent Psychiatric disorder according to the Minnesota International Neuropsychiatric Invetory (MINI – DSM-IV).

### Effect of treatment on immediate and delayed memory (primary outcome), verbal fluency and WM (secondary outcomes)

Immediate and delayed recall of episodic memory assessed with the Rey Auditory-Verbal Learning Test (RAVLT), orthographic and semantic category verbal fluency assessed with the Controlled Oral Word Association Test (COWAT), the Paced Auditory Serial Addiction Test (PASAT) and short-term and WM assessed with the Forward (FDS) and Backward Digit Span (BDS) were compared between treatment groups. Table 2 reports the results.

**Table 2:** Independent *t*-Tests Between Active and Sham-tDCS + WMT Groups for the Differences (Deltas) of Cognitive Scores from Pre to Post-Treatment (n = 39).

Cognitive Measures	Active-tDCS + WMT (n = 19) M(SD)			Sham-tDCS + WMT (n = 20) M(SD)			Between-groups P values for Δs
	Pre-treatment	Pos-treatment	Delta (Δ)	Pre-treatment	Pos-treatment	Delta (Δ)	
Δ COWAT Orthographic	28.80(11.24)	34.05(9.65)	23.46(27.94)	31.30(10.88)	34.31(11.88)	10.74(19.70)	0.11
Δ COWAT Semantic	16.80(6.07)	18.90(5.85)	14.08(23.78)	17.55(5.66)	17.26(5.15)	0.95(16.32)	0.52
Δ RAVLT A1	6.50(1.67)	9.20(2.56)	45.55(40.60)	6.80(2.09)	8.26(1.96)	25.52(35.96)	0.11
Δ RAVLT A1_A5	50.10(10.29)	58.40(8.74)	17.30(15.01)	45.70(10.15)	53.26(9.21)	18.03(15.33)	0.88
Δ RAVLT A7	9.65(2.88)	12.20(2.30)	28.37(23.67)	8.70(2.57)	10.94(2.73)	25.23(24.13)	0.68
Δ RAVLT Recognition	13.30(1.78)	14.10(1.20)	6.17(13.21)	13.50(1.27)	13.89(1.41)	2.72(10.75)	0.37
Δ PASAT	28.73(13.04)	33.50(12.61)	18.42(26.93)	28.15(11.30)	32.72(12.10)	18.21	0.98
Δ FDS	6.90(1.74)	6.45(1.50)	−4.39(18.29)	6.90(2.29)	6.00(1.69)	−9.62	0.45
Δ BDS	4.60(1.35)	5.05(1.82)	15.66(49.17)	4.30(1.62)	4.73(2.02)	12.36(39.61)	0.81

Data presented as mean (M) and standard deviation (SD).

Notes. Δ = deltas; COWAT = Controlled Word Association Test; PASAT = Paced Auditory Serial Addiction Test; RAVLT = Rey Auditory Verbal Learning Test; FDS = Forward Digit Span; BDS = Backward Digit Span. P-value is the comparison of the deltas. Significance level was P < 0.05.

### Effect of treatment on primary and secondary outcomes considering BDNF levels and years of education as covariates

The regression analysis exploring variables that influence BDNF levels showed presence of psychiatric diagnosis (B = −3.64; P < 0.007) and number of medications (B = 9.30; P < 0.019) significantly explained BDNF levels (R² = 0.22). Thus, the adjusted values of BDNF were used for the following analyses. The MANCOVA revealed that treatment had a significant influence in the model [Wilk’s Lambda = 0.488; F(9,24) = 2.80; P = 0.021; η² = 0.512], as well as the interaction term treatment*BDNF adjusted index [Wilk’s Lambda = 0.264; F(9, 24) = 2.52; P = 0.005; η² = 0.486]. Years of education was non-significant (P = 0.069). The influence of the factor treatment and covariates (years of education and BDNF adjusted index) together for each cognitive score is presented in Table 3. Accordingly, the model was significant for verbal fluency measures (COWAT orthographic and semantic), immediate recall (RAVLT A1_A5) and short-term memory (with the FDS score), considering Δs.

**Table 3:** Analysis of Covariance (ANCOVA) Models for the Association of Treatment, years of education and BDNF adjusted index on Deltas of Cognitive Scores (n = 39).

*Cognitive Measures*	Type III Sum of Squares	*df*	Mean Square	F	*p*	η²_partial_
Δ COWAT Orthographic	6257.07	4	1564.27	3.26	**0.024**	0.29
Δ COWAT Semantic	4241.91	4	1060.48	3.05	**0.031**	0.28
Δ RAVLT A1	8996.26	4	2249.07	1.47	0.233	0.16
Δ RAVLT A1_A5	2534.90	4	633.72	3.42	**0.020**	0.30
Δ RAVLT A7	6695.88	4	1673.97	1.80	0.152	0.18
Δ RAVLT Recognition	151.84	4	37.96	0.23	0.921	0.03
Δ PASAT	3699.63	4	924.91	1.50	0.225	0.16
Δ FDS	5716.00	4	1429.00	4.01	**0.010**	0.33
Δ BDS	12014.31	4	3003.58	1.54	0.213	0.16

Notes: df = degrees of freedom; Δ = deltas; COWAT = Controlled Word Association Test; PASAT = Paced Auditory Serial Addiction Test; RAVLT = Rey Auditory Verbal Learning Test; FDS = Forward Digit Span; BDS = Backward Digit Span. Statistics refer to the Corrected Model, with Treatment (active and sham-TDCS + WMT) and Treatment*BDNF adjusted index as factors and years of education as covariate. Significance level was P < 0.05.

In Table 4, we investigated in depth how group factor and covariates are associated with the cognitive scores, using univariate linear regression analyses as parameters estimates. As can be seen, belonging to the a-tDCS + WM training group was associated with an increased change in orthographic verbal fluency and immediate recall, independently of the educational and the BDNF level. Year of study was negatively associated with changes in orthographic verbal fluency score. Moreover, BDNF adjusted index correlated negatively with changes in immediate memory recall for the active tDCS and positively with the sham-tDCS group, while it correlated negatively with changes in FDS for the sham tDCS group only.

**Table 4:** Univariate Linear Regression Models for the Effects of Treatment Groups (Active and Sham-tDCS + WMT), Years of education (as a Covariate) and the Interaction Treatment*BDNF on Deltas of Cognitive Measures (n = 39).

Dependent Variable	B	SEM	F	P
**Δ COWAT Orthographic**				
Intercept	1.12	28.32	0.04	0.969
Active tDCS	75.36	35.77	2.10	**0.043**
Sham tDCS^a^	.	.	.	.
Education (years)	−2.43	1.01	−2.39	**0.022**
Active tDCS*index BDNF	−0.91	0.77	−1.18	0.244
Sham tDCS*index BDNF	1.54	0.90	1.27	0.211
**Δ COWAT Semantic**				
Intercept	−17.56	24.14	−0.72	0.472
Active tDCS	45.25	30.49	1.49	0.145
Sham tDCS^a^	.	.	.	.
Education (years)	1.45	0.86	1.68	0.103
Active tDCS*index BDNF	−0.93	0.65	−1.42	0.164
Sham tDCS*index BDNF	−0.01	0.77	−0.01	0.999
**Δ RAVLT A1_A5**				
Intercept	−6.37	17.62	−0.31	0.720
Active tDCS	74.29	22.26	3.33	**0.002**
Sham tDCS^a^	.	.	.	.
Education (years)	−0.80	0.63	−1.28	0.210
Active tDCS*index BDNF	−1.39	0.47	−2.90	**0.007**
Sham tDCS*index BDNF	1.15	0.53	2.04	**0.049**
**Δ FDS**				
Intercept	78.12	24,430	3,198	**0.003**
Active tDCS	−46.00	30.85	−1.49	0.146
Sham tDCS^a^	.	.	.	.
Education (years)	−1.49	0.87	−1.31	0.199
Active tDCS*index BDNF	−0.77	0.66	−1.16	0.251
Sham tDCS*index BDNF	−2.61	0.78	−3.35	**0.002**

Notes: df = degrees of freedom; SEM = standard error of the mean; COWAT = Controlled Word Association Test; RAVLT = Rey Auditory Verbal Learning Test; FDS = Forward Digit Span.

^a^Comparative group, to which values are referenced to. Significance level was P < 0.05.

### Associations between BDNF adjusted index and episodic memory scores

**Figure 1:**
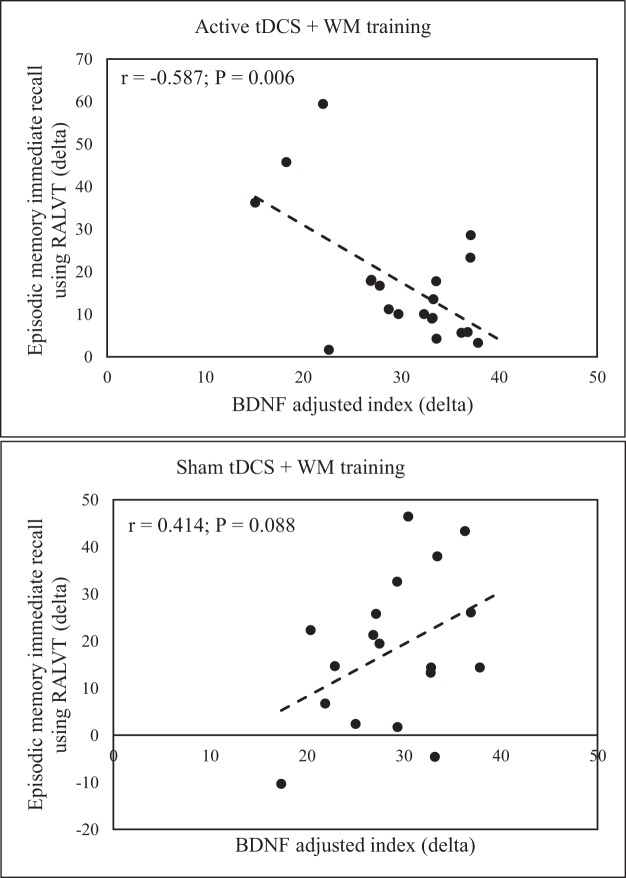
Scatter plots indicating the Pearson (r) correlations between changes in episodic memory immediate recall assess with the Rey Auditory Verbal Learning Test (RALVT) and changes in BDNF adjusted index.

Figure 1 depicts this interaction, where the correlation was only significant for the active tDCS group.

## Discussion

The present study aimed to test if a treatment tDCS coupled with a WM cognitive task would have additive effects that benefit memory, attention and executive functions for patients with FM when compared to cognitive training alone. In fact, data suggests this was the case regarding the higher increase in immediate memory capacity and verbal fluency after active treatment compared to sham. Interestingly, the effect of active tDCS on the short-term memory was partially dependent on baseline levels of serum BDNF. This neurotrophin was associated with changes in RAVLT A1_A5 score only for the active tDCS. Nevertheless, BNDF mediated changes in short-term digit span memory only for the placebo group. Also, years of education did not influence significantly the effect of interventions.

Our findings are congruent with other studies that found a better effect of tDCS combined with a cognitive training on WM and other cognitive performances^17–19^. Recent studies have showed that tDCS combined with a cognitive training task is more efficient to improve the pain threshold in FM compared to sham stimulation^20^. Particularly, in Silva *et al*.’s (2017)^13^ study, anodal tDCS applied on the DLPFC coupled with a training task for inhibitory control (a Go No-Go task) improved the executive and orienting attentional networks performance after a single session. In addition, previous researchers have found that for healthy volunteers the DLPFC anodal stimulation combined with an adapted verbal n-Back Task for training improved recall performance of word pairs^21–23^. It is possible that the impact of treatment observed in our study may also be associated with the lateralization of verbal material processing. This hypothesis is plausible since our sample comprises right-hand subjects only, which have mostly formal aspects of language being processed by the left-hemisphere^23^. Considering this rationale, the stimulus modality (verbal and visual) of the WM training task may interacts in a particular way with the site of the stimulation (left or right DLPFC). For example, the tDCS task-congruent intervention had a stronger and long-lasting enhancement of cognitive outcomes^23^. However, the effect reported by some authors^23,24^ was observed in healthy subjects. Our study comprised only verbal tasks for cognitive assessment. So, further studies would be necessary to test the hemisphere lateralization hypothesis. Using a similar methodology of the present study, Elmasry, Loo, and Martin (2015)^25^ concluded that ten sessions of online tDCS combined with a cognitive training (Dual n-Back Task) were not able to change neither WM nor executive function measures significantly. However, active tDCS improved the Dual n-Back discrimination ability^25^. This finding is quite similar to our univariate data, where no significant difference between groups was found. So, it is possible to argue that baseline factors associated with the central neurophysiological state are likely to influence this sort of treatment. This argument is supported by a growing body of evidence suggesting that tDCS produces a state-dependent impact when considering cognitive outcomes^26^. In this line, we found that a factor closely related to neuroplasticity state (BDNF) have influenced the effectiveness of treatment.

Baseline BDNF had a relevant effect on short-term memory indices. The RAVLT A1_A5, which assess the cumulative short storage capacity after a word list is presented five times to the patient, is a renowned instrument for episodic memory assessment^27^. Our data suggests tDCS induced a higher increase in this function from pre to post-intervention compared to sham. However, this effect was only significant when the interaction term, considering BDNF levels, was included. According to Table 2 *t-*tests, changes due to active or sham treatment have roughly the same magnitude. But when the interaction between Treatment and BDNF is considered (Table 4), it becomes clearer this neurotrophine had an opposite effect for the groups. Figure 1 illustrates that higher levels of BDNF at baseline assessment were associated with smaller changes from pre to post-intervention.

We expected that higher BDNF serum level would be related with better performance on memory tests. There is some literature indicating a positive relation between BDNF level with verbal memory and learning capacity in healthy subjects^28,29^. Moreover, there is evidence that the volume of the left hippocampus mediates the association between BDNF and spatial memory^30^. It should be considered FM is known as a syndrome that comprises a central sensitization process associated with higher levels of BDNF compared to controls^8^. Simultaneously, higher levels of BDNF have been related to both higher pain scores and disability in FM^31^. Therefore, especially for this population, and perhaps other similar pain syndromes, higher levels of this neurotrophin may impact negatively on the tDCS effect, leading to smaller changes in cognitive outcomes. This effect occurs in the hippocampus region, which is central to memory processes, such as consolidation^32^. BDNF is also widespread in central and peripheral nervous system, and present in many neural systems^33^ and its concentration may affect each neural network differently. Therefore, it seems plausible that in patients with FM, high levels of BDNF are not only associated with pain increase and maintenance but may also may be associated with an adverse neurophysiological environment for therapeutic approaches. Another measure of immediate recall evaluated here is the FDS. The BDNF was inversely correlated with the change in this test only for the sham group. Considering that no simple effect for treatment was found, we deductively concluded that a higher BDNF level at baseline could reduce the changes from pre to post-intervention. However, it remains unclear yet why this relationship was observed only for the sham group. First point to be raised concerns the differences between tasks. Despite evaluating similar recall abilities in both cases, RAVLT involves meaningful stimuli (words), learning curves (by repeating the stimuli), a free-order recall method and complex associative strategies, whereas FDS is an auditory test that classically measures phonological components of short-term memory^34^. This idea may help to understand the different role of BDNF for the RAVLT A1_A5 score for the sham group, which had a positive relation. When we consider the associations between BDNF and cognitive measures for the sham group, it should be highlighted these patients did received an intervention, a cognitive training. Besides, it should be considered that serum BDNF accounts only partially for the central nervous system concentration of the neurotrophin^35,36^.

We have also found effects of our treatment in other cognitive systems, and that was independent of BDNF levels. The COWAT is a measure of verbal fluency and covers a wide range of cognitive functions, including verbal ability and executive control^37^. Some authors reviewed cognitive processes evaluated with verbal fluency tasks. They suggest category fluency tasks, such as the semantic COWAT, reflect better the verbal ability, while letter fluency, on which our orthographic test is based, reveals more executive aspects^38^. Semantic verbal fluency is associated with more anterior-ventrally localized networks of the frontal cortex, while letter fluency is located more posterior-dorsally. Thus, it is plausible to consider that a DLPC anodal stimulation combined with a WM task that equally recruits the DLPFC area (apart from other regions, see Constantinidis and Klingberg 2016)^39^ would have a more salient impact over executive functions, than language-related functions. Patients with FM are known to have executive attention and WM difficulties^40^ what indicates a more clinically relevant effect of our treatment. On the other hand, the diffuse effect of tDCS should not be neglected, which means DLPFC stimulations may increase excitability in various regions of the frontal lobe. Because fluency tasks have time restrictions, higher general processing speed associated with increased excitability would benefit the active group.

In overall, the effect of tDCS observed in the current study may suggest that the active-tDCS combined with a WMT induced an increase functioning of the inhibitory system. This hypothesis might be plausibility if we considered that the FM is the prototypical syndrome of CSS^41^, which encompasses an impaired function of neurons and circuits in nociceptive pathways, with an increase in either membrane excitability and synaptic efficacy, and reduced inhibition^42^. In fact, the neurobiological mechanism of FM^28^ involves an imbalance between excitatory and inhibitory systems, by a dysfunction in the GABAergic and glutamatergic pathways^43^. In this sense, therapeutic use of active-tDCS may induce long-lasting after-effects. It was found long-term potentiation and depression and involvement of NMDA-receptor channels related to the tDCS effects, as well as domaminergic and cholinergic systems^44^. The stimulation is able to change the neuronal calcium influx, protein synthesis, blood flow, the level of brain oxygenation. The results can, however, differ between healthy and individuals with some central nervous system dysfunction, such as FM patients^45^.

Also, the present study represents progress to the question of non-invasive treatment in FM patients about transfer effects. As we found performance enhancements in functions other than WM tasks, transfer effects to other cognitive processes are plausible to be considered. RAVLT, COWAT, and FDS measure functions other than WM. However, this idea should be regarded cautiously, because we have not found effects for PASAT or backward digit span scores, which measure different aspects of WM^40,46^. Another limitation of our study was that the Dual n-Back Task used for training purposes is a highly demanding task, especially for older patients not familiarized with the computer. Even considering the adaptive version, starting at 1-back and increasing according to accuracy performance, none of the patients was able to achieve more than 2-back WM load. This task may have exhausted the limits of WM and cognitive processing, not allowing performance gains. These inferences are also limited due to the lack of a healthy control group and a sham cognitive training. Also, we had a sample of women, which limits our conclusions to this gender, although it should be highlighted that FM has a higher prevalence in females^1^.

Overall, our results highlight two important conclusions. First, eight session of anodal tDCS over the left DLPFC combined with WM training has a modulatory effect on short-term memory capacity and verbal fluency after active treatment compared to sham stimulation. The secondary effect BDNF had a relevant effect in our model when we consider short-term memory indices. Also, these findings suggest that the effects of tDCS combined with a WM training relation to transfer effects to other cognitive processes are plausible to be considered.

## Methods

### Design, setting and participants

The methods and results sections are reported according to the CONSORT guidelines. All subjects provided written informed consent before participating in this randomized, double blind, sham-controlled, two arm parallel design with allocation ratio of 1:1. The study was approved by the Research Ethics Committee at Hospital de Clínicas de Porto Alegre (HCPA) (Institutional Review Board IRB 140369). The current controlled trial is registered at Clinical Trial (No. NCT02880917; End date: August 26, 2016).

We recruited 40 outpatients of the HCPA, all women aged between 18 and 65 years-old. They were invited via advertisement to participate from November 2015 to July 2017. Sample size was calculated based on previous with 0.25 effect size compare the effect of active tDCS and sham with alpha level of 0.05 and 80% power. FM was diagnosed according to American College of Rheumatology criteria^47^. Subjects were required to have a score at least 50 mm on the 0–100 mm visual analogue scale for pain during most of the days over the last three months^48^. Subjects were allowed to remain on analgesic medications, including drugs for which they were refractory, and these medications could not be adjusted during the study. Major depressive disorders were accepted as secondary to FM. Subjects with history of substance abuse or evidence of other pain-related disorder were excluded. Females pregnant, in breast-feeding or with a history of neurologic or oncologic disease and ischemic heart disease, kidney or hepatic insufficient were also excluded.

### Intervention

The intervention of tDCS (TCT, Hong Kong, China) combined with a cognitive training task was applied for eight consecutive days (working days). tDCS was delivered using the anode electrode positioned over the left DLPFC (F3 according to the 10–20 system for EEG) and the cathode electrode at right supraorbital region (Fp2). The electrodes were placed into–35 cm^2^ sponges soaked in saline solution for better current conductivity. Rubber bandages were used to hold the electrodes in place for the duration of stimulation. The active-tDCS condition, a constant current of 2 mA was applied for 20 min. For sham stimulation the electrodes were placed in the same position, but the stimulator was turned off after a ramp-up of 30 s of stimulation^49^. To evaluate the safety of tDCS, we used questionnaire based on previously reported adverse events.

**Figure 2:**
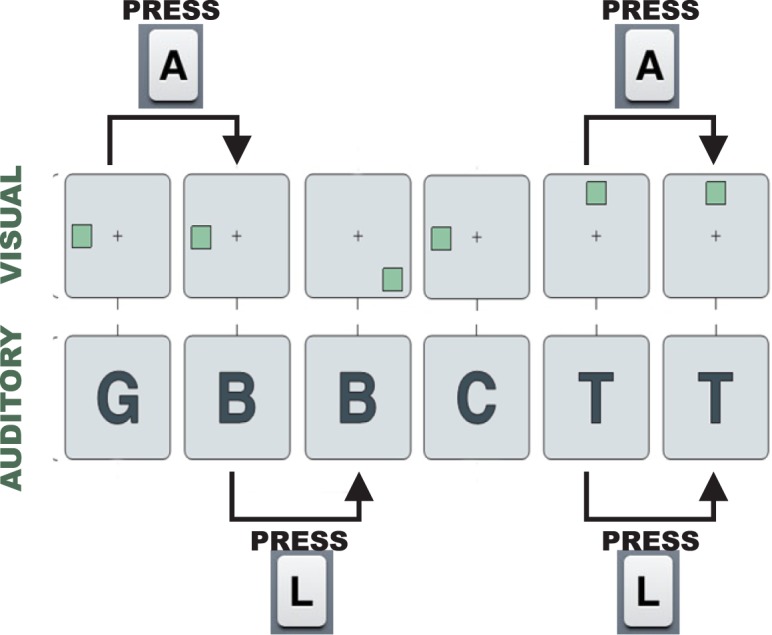
The Dual n-Back Task scheme.

The cognitive training consisted of an online application of a Dual N-Back task^50^. A laptop (15 inches screen, distance of ~60 cm ahead) with software E-Prime 2.0 Standard presented two types of stimuli, simultaneously. Visual stimuli were green squares presented in eight different positions, and auditory stimuli presented binaurally via headphones. Patients had to decide for each trial if the stimuli were the same as n-trials before (memory workload), by pressing the keyboard “A” button for visual and “L” for auditory (and do not press any button when none of the alternatives apply). The task had 20 blocks with 20 trials each, of which 10 were “no target”, 4 were “visual target only”, 4 “auditory target only” and 2 “dual target”, and had duration of about 25 minutes. The memory workload information was presented in the beginning of each block, and a feedback (percentage of correct responses) was presented in the end of each block. Because of the adaptive nature of the cognitive training, workload level increased when the previous block had 90% correct responses or higher and decreased when less than 70% was achieved. (Fig. 2).

### Randomization

Before the recruitment phase, the randomization was generated using a computer system by researchers who did not administer the intervention. They put the sequence in separately opaque sealed envelopes. The simple randomization method was applied, with patients assigned to the one of the two groups with a rate of 1:1.

### Blinding

Envelopes containing the patients’ protocol numbers were opened by an auxiliary researcher, who also programmed the tDCS device for active or sham stimulation.Allocation concealment was assured by intervention being assigned only after enrollment.Furthermore, to assess whether blinding was effective, at the end of the experiment subjects were asked to guess whether they had received a-tDCS or sham and to rate their confidence level using a 5-point Likert scale.

### Baseline instruments and assessments

All tests used have been validated for the Brazilian population. At the baseline, the instruments used were: Pittsburgh Sleep Quality Index^51^ to assess the sleep quality; Beck Depression Inventory-II (BDI-II)^52^, for the assessment of depressive symptoms; The Brazilian validated version of the Fibromyalgia Impact Questionnaire (FIQ)^53^, to assess quality of life of FM patients; the Brazilian Portuguese version of the Pain Catastrophizing Scale (BP-PCS)^54^, for the catastrophic thinking, State-Trait Anxiety Inventory (STAI) for the assessment of Anxiety^55^; Brazilian Profile of Chronic Pain: Screen (B-PCP:S)^56^ to characterize functional limitations related to severity of pain, emotional stress and pain interference in life; Pain level was assessed with a visual-analogue scale (VAS); Mini-International Neuropsychiatric Interview (M.I.N.I.)^57^ to detect psychiatric disorder; Medical comorbidities and demographic data were assessed using a standardized questionnaire. Heat pain threshold; Heat pain tolerance; BDNF marker of plasticity.

### Outcomes and instruments of assessment

The primary outcome was the performance of the Rey Auditory-Verbal Learning Test (RAVLT). The second outcomes were performance of the Paced Auditory Serial Addiction Test (PASAT)^58^, Controlled Oral Word Association Test (COWAT), Forward Digit Span (FDS), Backward Digit Span(BDS) and serum level of BDNF.

#### The Rey Auditory-Verbal Learning Test

RAVLT is a test for the evaluation of episodic memory, with components related to short- and long-term memory and recognition. The 15 words of the test were read slowly, and patients were asked to recall them regardless of the order (A1). The same procedure was repeated in the following steps A2, A3, A4 and A5. A second list of words (B1) was then applied and patients were asked to evoke them immediately. Afterwards, patients were asked to recall the first list (A6). About 20 to 30 minutes later, patients had to recall the words from the first list (A7) once more. Finally, a list of 50 words was presented and patients should judge whether the word belonged or not to the first list^27^.

#### Controlled Oral Word Association Test (COWAT)

Involves word fluency for two categories: orthographic and semantic. In the orthographic category, patients were instructed to say aloud as many words having F, A or S as the first letter as possible in 1 minute. In the semantic category, subjects were instructed to say aloud as many animal names as possible in 1 minute^59^.

#### Forward and backward digit span

The test consists of arrays of algorisms, presented each a time, with a gradual increase in the array (starting with two digits) for direct order (eight arrays; FDS) and for reverse order (seven arrays; BDS). Patients were instructed to recall the numbers immediately and in a serial order (FDS) or in inverse order (BDS). FDS was applied first, followed by BDS^46^. The maximum score is 16 points for the FDS and 14 points for the BDS.

#### Paced Auditory Serial Addiction Test (PASAT)

It evaluates sustained and divided attention and working memory. In this test, the stimuli are numbers from one to nine, presented in random and predetermined sequence. The task was to perform the sum of the numbers presented, two by two, disregarding the result of the calculation. The test started by displaying the numerical sequence every 3 seconds. It comprises equivalent versions A and B. In this project we use version A in the first assessment phase and B in the second one to prevent habituation. Maximum number of correct answers is 60 in each version.

#### Serum levels of brain-derived neurotrophic factor (BDNF)

Serum BDNF was determined by the Enzyme-Linked Immunoabsorbent Assay (ELISA) using a ChemiKine BDNF Sandwich ELISA Kit, CYT306 (Chemicon/Millipore, Billerica, MA, USA). The lower detection limit of the kit is 7.8 pg/mL for BDNF.

### General Procedure

**Figure 3:**
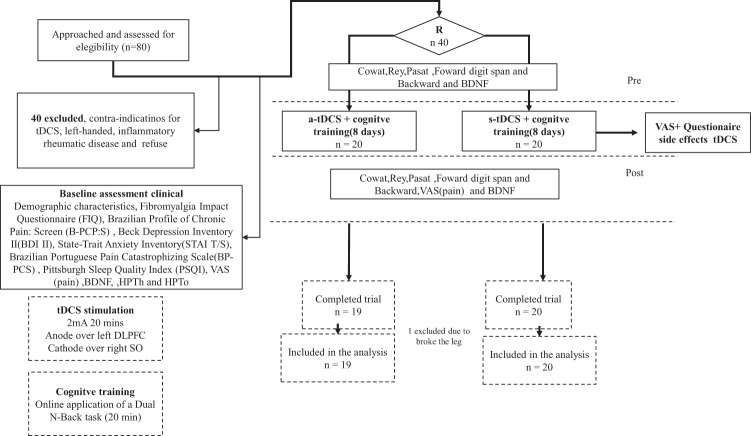
Flowchart showing recruitment and progress through the study. Controlled Word Association Test (Cowat); Rey Auditory Verbal Learning Test (RAVLT); Paced Auditory Serial Addiction Test (PASAT); Brain-derived neurotrophic factor(BDNF); Heat pain threshold (HPTh) and tolerance (HPTo).

Participants initially volunteered by signing the consent form. Following this, they responded to the baseline assessments and were checked for necessary exclusion criteria. Then, they were randomly allocated to one of the experimental groups, either receiving sham or active stimulation.Measures of working memory through the Dual N-back were obtained during the tDCS. Figure 3 presents the flowchart of the study.

### Statistical plan of analysis

Descriptive analysis were performed using mean, standard deviation and frequency. Inferential tests for demographic and clinical measures, as well as for the cognitive outcomes, were based on independent samples t-Tests for continuous variables and Chi-Squared or Fisher’s tests for categorical variables. To avoid baseline differences, we used deltas (Δ) based on the mean differences calculation [(post-test – pre-test)/pre-test] for cognitive outcomes. In order to test the influence of BDNF levels as a modulator for the treatment’s effect, we used a multivariate analysis of covariance (MANCOVA) for the cognitive scores as dependent variables. Due to many factors may influence serum BDNF level, we have adjusted its value in a linear regression model with stepwise method, which tested the influence of age, number of medications and frequency of medication use, presence of psychiatric diagnosis and baseline depressive symptoms (using the BDI-II) and functional incapacity (using the B-PCP:S). We also included years of education as a covariate because raw scores were used (instead of standardized scores), which may be affected by educational level. We tested single effects for treatment (active-tDCS + WM training and sham-tDCS + WM training) and years of education, and interaction between treatment and BDNF adjusted index, followed by Bonferroni correction and univariate linear regression analyses when significant main effects were found. Finally, for exploratory purposes, Pearson correlations between BDNF adjusted index and immediate memory recall scores were applied for each treatment group.

### Data availability

The datasets generated during and/or analyzed during the current study are available from the corresponding author on reasonable request.
